# Coexpression of invasive markers (uPA, CD44) and multiple drug-resistance proteins (MDR1, MRP2) is correlated with epithelial ovarian cancer progression

**DOI:** 10.1038/sj.bjc.6605185

**Published:** 2009-07-14

**Authors:** H Chen, J Hao, L Wang, Y Li

**Affiliations:** 1 Department of Gynecologic Oncology, Henan Tumour Hospital, 127 Dongming Rd, Zhengzhou, Henan 450008, China; 2 Faculty of Medicine, University of New South Wales, Kensington, NSW 2052, Australia; 3 Cancer Care Centre, St George Hospital, Gray St Kogarah, NSW 2217, Australia

**Keywords:** epithelial ovarian cancer, uPA, CD44, multiple drug-resistance proteins, metastasis

## Abstract

**Background:**

Invasion and metastases of cancer cells and the development of resistance to anticancer therapies are the main causes of treatment failure and mortality in cancer patients.

**Methods:**

We evaluated invasive markers of urokinase plasminogen activator (uPA) and CD44 and multiple drug-resistance (MDR) markers of MDR1 and MRP2 in four epithelial ovarian cancer (EOC) cell lines, primary tumours (*n*=120) and matched metastatic lesions (*n*=40) by immunofluoresence labelling. We correlated uPA and CD44 with MDR markers in primary and metastatic cells using confocal microscope. We also investigated the relationship of the expression of uPA, CD44 and MDR1 with various progression parameters.

**Results:**

The coexpression of uPA and CD44 with MDR markers was found in primary and metastatic cells. The overexpression of uPA, CD44 and MDR1 was found in most primary and matched metastatic lesions of EOC, and was significantly associated with tumour stage, grade, residual disease status, relapse and presence of ascites (*P*<0.05), but not with histology type (*P*>0.05).

**Conclusions:**

Our results suggest that the overexpression of uPA, CD44 and MRD1 is correlated with EOC progression; both uPA and CD44 are related with drug resistance during EOC metastasis and could be useful therapeutically.

Ovarian cancer is one of the most fatal malignancies. The standard treatment for advanced ovarian cancer is surgical removal of tumour, followed by platinum-based chemotherapy. Although patients may initially respond to chemotherapy, many still relapse, develop drug resistance and ultimately succumb to the disease (Bhoola and Hoskins, 2006). There is indirect evidence that the phenotypes between metastasis and multiple drug resistance (MDR) may be functionally linked. However, the relationship between them is still unclear.

Urokinase plasminogen activator (uPA) is a member of the serine protease family and is strongly implicated as a promoter of tumour progression in various human malignancies. By binding to uPA receptors (uPAR), uPA efficiently converts the inactive zymogen, plasminogen, into the active serine protease, plasmin, which then directly or indirectly cleaves extracellular matrix (ECM) components including laminin, fibronectin, fibrin, vitronectin and collagen (Andreasen *et al*, 1997). Plasmin can activate latent elastase and matrix metalloproteinases, potent enzymes that can also digest a variety of ECM components (Andreasen *et al*, 1997). Overwhelming evidence shows that the cell-surface-associated uPA–uPAR complex is causatively involved in tumour invasion and metastasis of many types of cancers by exerting multifaceted functions through either direct or indirect interactions with integrins, endocytosis receptors and growth factors (Andreasen *et al*, 1997; Sidenius and Blasi, 2003).

CD44 is a family of cell-surface glycoproteins that are expressed in a variety of human solid tumours, particularly those of gynaecological origin (e.g., ovarian cancers) (Naor *et al*, 1997; Kayastha *et al*, 1999), and is implicated in cell adhesion, motility and metastases (Naor *et al*, 1997). The gene that encodes CD44 contains 19 exons and is alternatively spliced, giving rise to many CD44 isoforms. All CD44 isoforms contain a hyaluronic acid (HA)-binding site in their extracellular domain, which serves as the major cell-surface receptor for HA (Underhill, 1992).

A major mechanism for drug resistance in cancer is energy-dependent efflux pumps that reduce intracellular accumulation. MDR1/P-glycoprotein (P-gp) called as ABCB1, a 170-kDa membrane phosphoglycoprotein encoded by the *mdr*1 gene (*MDR1*) located on chromosome 7q21, is a well-characterised member of energy-dependent drug efflux pumps (Germann, 1996). MRP2 is a member of the MRP/ABCC subfamily and also has an important function in the occurrence of the MDR phenotype in cancer cells (Kruh and Belinsky, 2003). It has been reported that MRP2 confers resistance to several other anticancer agents being non-platinum-containing drugs, including methotrexate, vinblastine and camptothecin derivatives (Cui *et al*, 1999).

A correlation between the expression of MDR1 and CD44 has been found in breast cancer cell lines, which showed that the two proteins colocalise within the cell membrane, that one protein directly influences the expression of the other and that a disruption of this interaction has profound effects on drug resistance, cell migration and *in vitro* invasion (Miletti-Gonzalez *et al*, 2005). Specifically, HA binding to CD44 is capable of stimulating MDR1 expression and drug resistance in breast tumour cells through ErbB2 signalling and PI3 kinase/Akt-related survival pathways (Misra *et al*, 2005). Bourguignon *et al* (2008) have recently shown that HA–CD44 interaction activates stem cell marker Nanog, Stat-3-mediated MDR1 gene expression and ankyrin-regulated multidrug efflux in breast and ovarian tumour cells. However, a direct link of phenotypes between drug resistance and invasion or metastasis in epithelial ovarian cancer (EOC) is still unclear.

The purpose of this study was to investigate whether a linkage exists between metastatic potential markers (uPA, CD44) and MDR proteins (MDR1, MRP2) during EOC progression. We found a colocalisation of uPA, CD44, MDR1 and MRP2 in primary and metastatic EOC cells lines. We showed that overexpression of uPA, CD44 and MRD1 is correlated with EOC progression. In addition, we further confirmed the colocalisation of uPA, CD44 and MDR1 in primary and metastatic lesions of EOC tissues. Our results suggest that uPA and CD44 are related to drug resistance and could be useful therapeutic targets for the prevention of the development of incurable, recurrent and drug-resistance EOC.

## Materials and methods

### Antibodies

The following antibodies were used: mouse anti-human uPA IgG_1_ (no. 394) (American Diagnostica, Greenwich, CT, USA); rabbit anti-human CD44 monoclonal antibody (MAb) (ab51037) and rabbit anti-human uPA (ab24121) polyclonal antibody (Abcam, Cambridge, UK); mouse anti-human CD44 and mouse anti-human IgG_1_-negative control MAbs (Dako, Glostrup, Denmark); rabbit anti-human MDR1 polyclonal antibody (sc-1517-R) (Santa Cruz Biotechnology Inc., Santa Cruz, CA, USA); mouse anti-humanMRP2 (M_2_III-6) MAb (Alexis Biochemicals, San Diego, CA, USA); and Alexa Fluor-488 goat anti-mouse IgG and Alexa Fluor-594 goat anti-rabbit IgG (Molecular Probes, Eugene, OR, USA).

### Cell lines and cell culture

The primary (OVCAR-3, A2780) and metastatic (SKOV-3, OV-90) EOC cell lines were obtained from American Type Culture Collection (ATCC, Rockville, MD, USA). All tissue culture reagents were supplied by Invitrogen Australia Pty Ltd (Melbourne, VIC, Australia), unless otherwise stated. OVCAR-3, A2780 and SKOV-3 cells were cultured in RPMI-1640 supplemented with 10% (v/v) heat-inactivated fetal bovine serum (FBS), 50 U ml^−1^ penicillin and 50 U ml^−1^ streptomycin. OV-90 cells were maintained in a 1 : 1 mixture of MCDB 105 medium (Sigma-Aldrich, St Louis, MO, USA) and 199 medium (Sigma-Aldrich), supplemented with 15% FBS, 50 U ml^−1^ penicillin and 50 U ml^−1^ streptomycin. All cell lines were maintained in a humidified incubator at 37°C and 5% CO_2_. Subconfluent cells that had been in culture for 48 h without a change of medium were harvested by gently rinsing flasks twice with Dulbecco's phosphate-buffered saline and then detached with 0.25% trypsin/0.05% EDTA in phosphate-buffered saline at 37°C. Cells were collected and resuspended in the appropriate buffer as described above.

### Immunofluorescence confocal microscopy analysis

To determine the cellular localisation of uPA, CD44, MDR1 and MRP2 in EOC cells, OVCAR-3, A2780, SKOV-3 and OV-90 cells were grown on glass coverslips (10^5^ cells) for 24 h. After washing with Tris-buffered saline (TBS) (pH 7.5), the cells were fixed on coverslips in ice-cold methanol for 10 min at room temperature (RT) and then incubated with 10% normal goat serum in TBS for 20 min to suppress the nonspecific binding of IgG. After washing once again with TBS, the cells were incubated with mouse anti-uPA (1 : 300 dilution) or rabbit anti-uPA (1 : 300 dilution), mouse anti-CD44 (1 : 50 dilution) or rabbit anti-CD44 (1 : 300 dilution), anti-MDR1 (1 : 300 dilution) and anti-MRP2 (1 : 50 dilution) antibodies for 1 h at RT on a shaking table and rinsed with TBS, followed by a 45 min incubation with Alexa Fluor-conjugated anti-mouse or anti-rabbit IgG (1 : 1000 dilution) for 1 h at RT. The stained cells were mounted on glass slides using glycerol (Sigma-Aldrich Pty Ltd, Castle Hills, NSW, Australia). Examination was performed with Confocal Microscope (FV 300/FV500 Olympus, Tokyo, Japan). Negative control slides were treated identically by isotype control MAbs or the primary antibody was omitted as a negative control.

### Patients and clinical data

A total of 120 primary EOC and 40 corresponding intraperitoneal metastatic lesions were obtained from the surgical pathology files of the Department of Pathology, Henan Tumour Hospital, China. All patients underwent primary surgery at the Department of Gynecological Oncology between 2001 and 2007. None of the patients had received chemotherapy before surgery. Clinical data were obtained by a retrospective review of the medical records. The study was approved by the Institutional Review Board, Henan Tumour Hospital. Tumours were staged according to the criteria of the International Federation of Gynecology and Obstetrics (FIGO) (Creasman, 1989). Details of the patients' characteristics are summarised in Table 1. Twenty normal ovarian specimens (controls) were obtained from early-stage cervical cancer patients with a mean age of 51±14 years (range, 40–70), who underwent surgery during the same period (Table 1). The criteria for tumour relapse were serum levels of CA125 >35 *μ*g ml^−1^, which continued to increase, with confirmation by B ultrasound, computed tomography, magnetic resonance imaging or positron emission tomography.

### Tumour tissue collection

Surgical specimens (EOC tissues and normal ovarian tissues) were fixed in 10% neutral-buffered formalin, routinely processed, whole-mount-embedded in paraffin and 4 *μ*m sections were collected. Haematoxylin and eosin (H&E)-stained sections were examined and tumour foci were identified, circled in ink and graded. All tissue specimens from primary tumours and metastatic lesions were verified by histology to confirm diagnosis, histological type and tumour grade (Dr QingKai Yu, Pathologist, Director of Department of Pathology, Henan Tumour Hospital, China).

### Immunofluorescence staining on EOC tissues

For the expression of uPA, CD44 and MDR1, sections including primary and metastatic tumours were deparaffinised in xylene and dehydrated in a graded series of alcohol (100, 95 and 75%) and rehydrated in TBS. Antigen retrieval was performed in a boiling citrate buffer (0.01 M, pH 6.0) for 20 min. Thereafter, the sections were incubated with normal goat serum (1 : 10 dilution) for 10 min and then incubated overnight with mouse anti-uPA MAbs no. 394 (1 : 100 dilution), mouse CD44 (1 : 50 dilution) and rabbit MDR1 (1 : 200 dilution) at 4°C. After washing with TBS, the slides were incubated with Alexa Fluor 488 goat anti-mouse IgG (1 : 1000 dilution) or Alexa Fluor 594 goat anti-rabbit IgG (1 : 1000 dilution) in the dark for 45 min at RT, and then mounted with Gleracol (Sigma-Aldrich). Negative control slides were treated identically, but isotype control MAbs (mouse anti-human IgG_1_) were used or the primary antibody was omitted as a negative control. The PC-3 prostate cancer cell line (uPA, CD44 and MDR1 positive) was used as a positive control.

For colocalisation of uPA, CD44 and MDR, sections including primary and metastatic tumours were incubated overnight at 4°C in primary mouse anti-uPA (no. 394) (1 : 100 dilution) and mouse CD44 (1 : 50 dilution) MAbs; and rabbit anti-uPA (1 : 100 dilution) and rabbit anti-human MDR1 (1 : 300 dilution) polyclonal antibodies, respectively. After washing with TBS, the sections were incubated in goat anti-mouse Alexa 488 (for mouse uPA and CD44) and goat anti-rabbit Alexa 594 (for rabbit uPA and MDR1) for 1 h at RT, and rinsed in TBS. Controls were treated identically, using nonspecific immunoglobulins (IgG_1_ or rabbit IgG) as negative control. The sections were examined using a Zeiss LSM 5 Pascal laser scanning confocal microscope and LSM 5 Pascal Image software. Multichannel excitation bleedthrough was minimised using fluorochromes with a large difference in peak excitation (488 and 594 nm, respectively). Examination was performed with a confocal microscope (FV 300/FV500 Olympus, Tokyo, Japan). Multitracking and sequential image capture was used to correct signal emission crosstalk between neighbouring channels, and the images were combined.

### Assessment of immunofluoresence staining results

Immunostaining results were assessed by staining intensity (0–3) for tested cancer cell lines and tissues. The criteria used for assessment were as follows: 0, negative or <25% tumour stained; 1+, weak or 25–50% tumour stained; 2+, moderate or 50–70% tumour stained; and 3+, strong or >75% tumour stained. Immunostaining was carried out independently by two experienced observers (HC and YL), and specimens were scored blindly and averaged. If results were discordant, differences were resolved by joint review and consultation with other experienced observers. For statistical analysis, EOC cases were divided into two groups: the low-expression group (LEG), composed of the − and 1+ groups, and the high-expression (overexpression) group (HEG), composed of the 2+ and 3+ groups.

### Statistical analysis

The associations between uPA, CD44 and MDR1 expression levels (LEG and HEG) and clinicopathological data were tested using the *χ*^2^-test. Comparison of staining intensity for uPA, CD44 and MDR1 between EOC tissues and normal ovarian tissues or between primary EOC and metastatic lesions was carried out using the *χ*^2^-test, where *P*<0.05 (two-tail) was considered significant. All statistical analyses were performed using GraphPad Prism 4.00 (GraphPad, San Diego, CA, USA).

## Results

### Expression and colocalisation of uPA, CD44, MDR1 and MRP2 in primary and metastatic EOC cell lines

**Figure 1:**
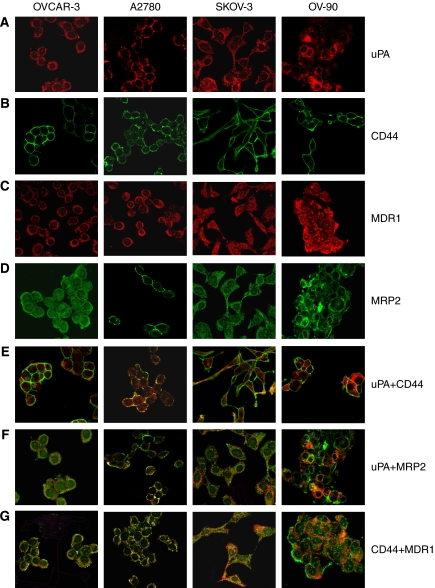
Coimmunolabelling of uPA, CD44, MDR1 and MRP2 in EOC. Representative confocal images of uPA and MDR1 (red; Alexa-594), and CD44 and MRP2 (green; Alexa-488) expression in EOC primary and metastatic cell lines are shown. Merged images, and red and green channels are shown separately. (**A**) uPA expression; (**B**) CD44 expression; (**C**) MDR1 expression; (**D**) MRP2 expression; (**E**) Colocalisation of uPA with CD44; (**F**) Colocalisation of uPA with MRP2; (**G**) Colocalisation of CD44 with MDR1. Magnification: **A**–**G** × 400.

**Figure 2:**
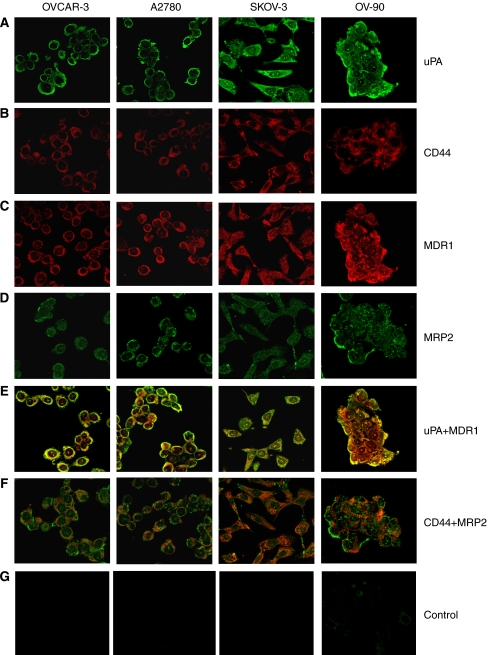
Coimmunolabelling of uPA, CD44, MDR1 and MRP2 in EOC. Representative confocal images of uPA and MRP2 (green; Alexa-488), and CD44 and MDR1 (red; Alexa-594) expression in EOC primary and metastatic cell lines are shown. Merged images, and red and green channels are shown separately. (**A**) uPA expression; (**B**) CD44 expression; (**C**) MDR1 expression; (**D**) MRP2 expression; (**E**) Colocalisation of uPA with MDR1; (**F**) Colocalisation of CD44 with MRP2; (**G**) IgG-negative control. Magnification: **A**–**G** × 400.

Immunofluorescence labelling results of EOC cells with uPA, CD44, MDR1 and MRP2 antibodies showed positive staining in all cancer cell lines (Figures 1 and 2). The staining intensities are summarised in Table 2. All EOC cell lines express medium to high levels of uPA, CD44, MDR1 and MRP2. Membrane expression was found in CD44 antibodies, whereas both membrane and cytoplasm expressions were found in uPA, MDR1 and MRP2 antibodies. The staining pattern is more homogeneous. Colocalisation of uPA/CD44, uPA/MDR1, uPA/MRP2, CD44/MDR1 and CD44/MRP2 was observed in all tested primary and metastatic cell lines (Figures 1 and 2).

### Expression of uPA, CD44 and MDR1 in primary EOC tissues and metastatic lesions

**Figure 3:**
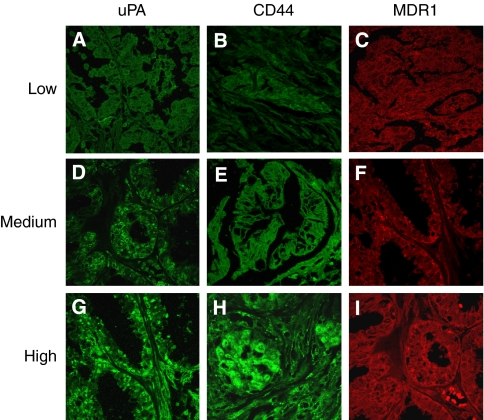
Immunofluoresence staining for uPA, CD44 and MDR1 in primary and metastatic EOCs. Representative images from different patients. Representative confocal images of uPA and CD44 (green; Alexa-488) and MDR1 (red; Alexa-594) expression in EOC primary and metastatic EOC tissues are shown. Low levels of uPA, CD44 and MDR1 are shown in primary EOC tissues (**A**–**C**), respectively. Medium levels of uPA, CD44 and MDR1 are shown in primary EOC tissues (**D**–**F**), respectively. High levels of uPA, CD44 and MDR1 are shown in metastatic EOC tissues (**G**–**I**), respectively. uPA immunolabelling is homogeneous and is generally seen on epithelial and stromal cells. Magnification: **A**–**I** × 400.

In primary EOC tissues, 88% (105 out of 120), 83% (100 out of 120) and 80% (96 out of 120) were positive to uPA, CD44 and MDR1 (1+ to 3+), respectively, whereas in the matched metastatic lesions, 90% (36 out of 40), 85% (34 out of 40) and 83% (33 out of 40) were positive to uPA, CD44 and MDR1 (1+ to 3+), respectively. Of the uPA-positive EOC sections, weak staining (1+) was found in 15% (16 out of 105) (Figure 3A), moderate staining (2+) in 45% (47 out of 105) (Figure 3D) and strong staining (3+) in 40% (42 out of 105) in primary tumours, whereas weak staining was found in 19% (7 out of 36), moderate staining in 44% (16 out of 36) and strong staining in 36% (13 out of 36) (Figure 3G) in metastatic lesions.

Of the CD44-positive sections, weak staining (1+) was found in 26% (26 of 100) (Figure 3B), moderate staining (2+) in 43% (43 of 100) (Figure 3E) and strong staining (3+) in 31% (31 of 100) in primary tumours, whereas weak staining (1+) was found in 20% (8 of 40), moderate staining (2+) in 35% (14 of 40) and strong staining (3+) in 30% (12 of 40) (Figure 3H) in metastatic lesions.

Of the MDR1-positive sections, weak staining (1+) was found in 21% (20 of 96) (Figure 3C), moderate staining (2+) in 44% (42 of 96) (Figure 3F) and strong staining (3+) in 35% (34 of 96) in primary tumours, whereas weak staining (1+) was found in 20% (8 of 40), moderate staining (2+) in 35% (14 of 40) and strong staining (3+) in 28% (11 of 40) (Figure 3I) in metastatic lesions.

No uPA, CD44 and MDR1 staining was found in normal ovarian tissues and negative sections from primary EOC tissues and metastatic lesions (data not shown). The staining intensity for uPA, CD44 and MDR1 in primary and metastatic lesions is summarised in Table 3, which shows the relationship of uPA, CD44 and MDR1 staining in two groups (primary and metastatic tumours). The concordance rate of uPA, CD44 and MDR1 was 87–90% in primary EOC and metastatic lesions. The primary tumours from patients with uPA, CD44 and MDR1-positive metastatic lesions also expressed uPA, CD44 and MDR1. The staining intensity was mostly moderate to strong in this group of patients.

The expression of uPA, CD44 and MDR1 was quite uniform in most tumours, and regions of heterogeneous staining were rarely seen. The expression of uPA and CD44 was mainly cell membrane associated; however, distinct positive cytoplasmic staining was also observed. Immunostaining of MDR1 was mainly cell cytoplasmic staining. In high-grade EOC (Grade 2 and 3), the most tumour stroma also showed a strong positive reaction for uPA, CD44 and MDR1 in primary tumours and metastatic lesions (data not shown).

### Correlation between clinical parameters and uPA, CD44 and MDR1 expression

Of the 120 EOC patients, 72% (87 of 120) relapsed with metastases. The median time to relapse was 42 months (range, 15–56 months). Overall, 52% of patients had residual tumours after first surgery and 82% of patients had ascites of more than 500 ml. Table 4 summarises the correlation among uPA, CD44 and MDR1 expressions in primary tumours with tumour grade, clinical stage, histological type, residual tumour after first surgery, relapse and ascites. The overexpression (HEG) of uPA, CD44 and MDR1 was correlated with relapse (*P*<0.01) and increased with the progression of EOC (tumour grade, *P*<0.01; clinical stage, *P*<0.05; residual tumour after first surgery, *P*<0.05; ascites, *P*<0.05). There was no correlation between the overexpression of uPA, CD44 and MDR1 and histological type.

### Coimmunolabelling of primary tumours and metastatic lesions with uPA, CD44 and MDR1 antibodies

**Figure 4:**
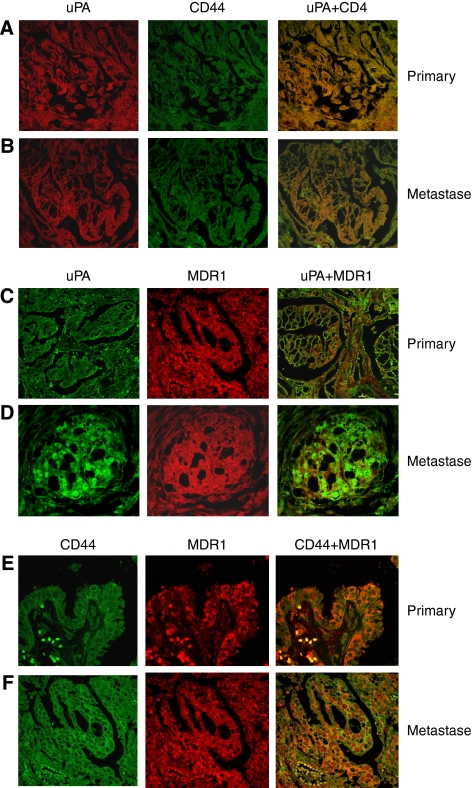
Coimmunolabelling of uPA, CD44 and MDR1 in EOC tissues. Representative confocal images of uPA (red; Alexa-594 or green; Alexa-488), CD44 (green; Alexa-488) and MDR1 (red; Alexa-594) expression in EOC primary tumours and matched metastatic lesions are shown. Merged images and red and green channels are shown separately. (**A**) Colocalisation of uPA with CD44 in primary EOC tissues; (**B**) Colocalisation of uPA with CD44 in metastatic EOC tissues; (**C**) Colocalisation of uPA with MDR1 in primary EOC tissues; (**D**) Colocalisation of uPA with MDR1 in metastatic EOC tissues; (**E**) Colocalisation of CD44 with MDR1 in primary EOC tissues; (**F**) Colocalisation of CD44 with MDR1 in metastatic EOC tissues. uPA immunolabelling is homogeneous and is generally seen on epithelial and stromal cells. Magnification: **A**–**F** × 400.

Colocalisation of uPA and CD44, uPA and MDR1, CD44 and MDR1 was further tested in primary tumours and matched metastatic lesions (*n*=40). Most of the tested samples were found to be coimmunolabelled with two different markers, although single staining in different samples was variable. The typical images from different tumours are shown in Figure 4. For coimmunolabelling of uPA with CD44 (Figure 4A and B) and of uPA with MDR1 (Figure 4C and D), uPA appears green, whereas CD44 and MDR1 appear red. For coimmunolabelling of CD44 with MDR1 (Figure 4E and F), CD44 appears green, whereas MDR1 appears red.

## Discussion

Invasion and metastases of cancer cells and the development of resistance to antidrug therapies are the main causes of morbidity and mortality from cancer. Proteolytic enzymes such as uPA system and CD44 have an active function in EOC metastasis. Drug-resistance proteins such as MDR1 and MRP2 are the best-known mediators of resistance to anticancer drugs, extruding many types of drugs from cancer cells, thereby conferring resistance to those agents. Although previous reports have shown that the presence of uPA, CD44 and MDR proteins was related to EOC progression and prognosis (Kayastha *et al*, 1999; Konecny *et al*, 2001; Penson *et al*, 2004), their expression and link between these two markers in primary EOCs and in metastatic microenvironment have not been fully investigated.

In this study, we examined the expression of uPA, CD44, MDR1 and MRP2 in EOC cell lines, in primary EOC, matched metastatic lesions and normal ovarian tissues using tissue bank, and investigated the link among these markers. High levels of uPA, CD44, MDR1 and MRP2 were observed in EOC cell lines, in advanced EOC specimens but not in normal ovarian tissues. Most of the metastatic lesions and matched primary cancer tissues expressed high levels of uPA, CD44 and MDR1. Colocalisation of metastatic markers (uPA, CD44) and MDR proteins (MDR1, MRP2) was also observed in EOC cells. To our knowledge, this is the first report investigating the link among uPA, CD44, MDR1 and MRP2 during EOC progression.

One of the interesting findings in this study is the colocalisation of uPA and CD44, uPA and MDR1, uPA and MRP2, CD44 and MDR1 or CD44 and MRP2 in primary and metastatic EOC cells. Kobayashi *et al* (2002) reported that CD44 stimulation by fragmented HA can activate MAP kinase (MAPK) proteins and upregulate the expression of uPA mRNA and protein in human chondrosarcoma cell line HCS-2/8, which supports the role of uPA as an invasion-promoting factor. Sheridan *et al* (2006) showed that breast cancer cell lines with a significant CD44+/CD24− sub-population express higher levels of the uPA gene associated with cancer invasion (Sheridan *et al*, 2006). uPA and uPAR are upregulated in tumours of various origins, wherein they have an important function in the development of invasive and chemoresistant cancer phenotypes (Gutova *et al*, 2007). The direct link between uPA and cancer stem cells (CSCs) or MDR proteins has not been reported. Gutova *et al* (2008) have recently shown that uPA can mediate human stem cell tropism to malignant solid tumours by chemotaxis and cell guidance. Here, we first showed the colocalisation of uPA and CD44 in primary and metastatic EOC cells, and the direct link between uPA and MDR proteins (MDR1 and MRP2). These results support the fact that uPA has a close relationship with CD44, and may be involved in the regulation of the expression of MDR1 and MRP2 during EOC metastasis.

CD44 is a marker of CSC (Dalerba *et al*, 2007). Zhang *et al* (2008) have recently identified a sub-population of CD44+CD117+ cells (ovarian cancer-initiating cells) from primary ovarian cancer tissues that are fully capable of a serial propagation of their original tumour phenotype in animals, suggesting that CD44 is involved in EOC progression. It was reported that hyaluronan–CD44 interactions regulate the expression of drug transporters, including P-glycoprotein (MDR1) and MRP2 (Misra *et al*, 2005). In this study, we found a direct link of uPA and CD44 with MDR proteins (MDR1and MRP2) in primary and metastatic EOC cells. In a parallel study, we compared metastatic prostate cancer cell line (DuCaP), which is negative to uPA, CD44, MDR1 and MRP2, with docetaxel drug-resistant prostate cancer cell line (PC-3M-Luc-MDR), which is strongly positive to uPA, CD44, MDR1 and MRP2. After treatment with a chemodrug (docetaxel), more DuCaP cells were killed compared with PC-3M-luc-MDR cells (unpublished data). These results suggest that uPA and CD44 may functionally regulate MDR1 or MRP2 expression and confer drug resistance to ovarian cells during cancer progression, further confirming the close link between invasive and metastatic markers (uPA and CD44) with MDR proteins (MDR1 and MRP2), and may have clinical significance for future therapy to target late-stage and drug-resistance ovarian cancer cells.

In ovarian cancers, a significant elevation of uPA levels is associated with prognosis and disease progression (Konecny *et al*, 2001). The increased expression of uPA mRNA is associated with the dedifferentiation of serous EOC from cystic to solid tumours (Borgfeldt *et al*, 2001). The levels of uPA in peripheral blood were higher in patients with EOC than in controls (Casslen *et al*, 1994). The tumour content of uPA was reported to be increased with loss of histological differentiation and also tended to increase in advanced FIGO stages in ovarian cancer (Borgfeldt *et al*, 1998). This study also indicates that the high level of expression of uPA was correlated with tumour grade, clinical stage, residual tumour, relapse and ascites. However, we found no difference in histological type. These results indicate that uPA has an important function in EOC development and metastasis.

Induction of CD44 expression has been noted during the development of EOC, but the issues of whether a high CD44 expression represents a relatively favourable prognosis (Ross *et al*, 2001) or an aggressive behaviour of the tumour and unfavourable prognosis (Kayastha *et al*, 1999), and whether CD44 has any prognostic significance (Berner *et al*, 2000), have remained controversial. Sillanpaa *et al*, (2003) reported that CD44 expression is related to well-differentiated, early-stage EOC and long survival of the patients, thus indicating a favourable prognosis in EOC. Kayastha *et al* reported that expression of CD44 is an independent predictor of survival in women with EOC. Afify *et al* (2006) reported that the expression of CD44s and CD44v5 is more common in stage III than in stage I serous ovarian carcinomas and suggested a role for CD44 and stromal HA in the dissemination of EOC. The large variation of the CD44 expression from study to study may be attributed to the different methodologies used in the assessment of CD44 expression or to the different stages of ovarian cancer in the analysis. In this study, most of our samples were from late-stage EOC and we found that a high level of expression of CD44 was correlated with tumour grade, clinical stage, residual tumour, relapse and ascites, but with no difference in histological type. Our results support the fact that CD44 is involved in EOC progression and metastasis.

Development of MDR1-mediated drug resistance results in failure of treatment in cancer. MDR1 is expressed in stem cells and CSCs, and thus is speculated to not only act as transporters pumping antitumour drugs out of cells (van Herwaarden *et al*, 2003) but to also play a role in the maturation and differentiation of these stem cells (Hadnagy *et al*, 2006). Xing *et al* (2007) recently reported that knock down of P-glycoprotein reverses taxol resistance in ovarian cancer multicellular spheroids. An increased expression of MDR1 was found to be associated with an unfavourable prognosis of ovarian cancer in some studies (Materna *et al*, 2004; Penson *et al*, 2004), but not in others (Yokoyama *et al*, 1999; Ozalp *et al*, 2002). These inconsistent results suggest that it is necessary to further investigate the correlation of MDR1 expression with ovarian cancer prognosis using a relatively large number of ovarian cancer tissues before chemotherapy. The large variation of MDR1 expression from study to study may be attributed to the different methodologies used in the assessment of MDR1 expression or to the different stages of ovarian cancer in the analysis. In our study, we analysed only primary tumour samples in the late stage of patients before any drug treatment. Our results demonstrated that a high level of expression of MDR1 was correlated with tumour grade, clinical stage, residual tumour, relapse and ascites, but with no difference in histological type. Our results support the fact that MDR1 is also involved in EOC progression and metastasis.

In this study, we found an overexpression of uPA, CD44 and MDR1 and a colocalisation of uPA and CD44, uPA and MDR1, CD44 and MDR1 in cancer cells and stromal cells from most primary tumours and matched metastatic lesions, and further confirm the finding in EOC cell lines. No change in uPA, CD44 and MDR1 expression was observed during the metastatic process, whereas others have observed a downregulation of CD44 during tumour progression in mice (Yeo *et al*, 1996) and in human ascitic tumour cells (Ross *et al*, 2001). These results suggest that CD44 expression may be regulated in different microenviroments during cancer metastasis. Our results indicate that overexpression of uPA, CD44 and MDR1 may involve EOC metastases and that cancer clones that escape from primary tumours do not lose these antigens. The functional interaction between CD44 and P-glycoprotein is one step in a complex molecular organisation that results in the concomitant phenotype of MDR1, in increased cell migration, *in vitro* invasion and metastasis (Miletti-Gonzalez *et al*, 2005); moreover, the colocalisation of uPA with CD44 or MDR1 was found in this study, suggesting that it is possible that both uPA and CD44 concomitantly regulate MDR1 expression during EOC for drug resistance. The exact mechanism in this regulation in EOC metastasis is still unclear and needs to be investigated in future study. Given that uPA and CD44 colocalise with MDR1-positive cells in EOC samples, it could be a useful therapeutic target for therapy in this disease to overcome drug resistance in the late stage of metastatic cancer. In our human tissue experiments, we did not examine MRP2 expression because this anti-MRP2 MAb does not work on paraffin-embedded sections. The expression of MRP2 in primary and metastatic EOC cell lines correlated with invasive markers (uPA and CD44), suggesting that MRP2 also has an important function in EOC metastasis and drug resistance. Targeting MRP2 is another option for late-stage and drug-resistant EOC disease.

We have shown that treatment with ^213^Bi-labelled PAI2 (targeting membrane-bund uPA) inhibits single EOC cells and spheroid growth *in vitro* (Song *et al*, 2006). Recently, a uPA-derived peptide, A6, which in animal models reduced tumour growth, metastasis and angiogenesis, alone or in combination with other therapies, was evaluated in a phase I clinical trial in patients with gynaecological cancers, especially OC. This study showed the safety of A6 and some clinical potential (Berkenblit *et al*, 2005). Banzato *et al* (2008) have recently reported that a paclitaxel-hyaluronan bioconjugate (ONCOFID-P) interacted with CD44, could target IGROV-1 and OVCAR-3 xenografts after i.p. administration and show promise in future clinical trial. A combination therapy targeting uPA and CD44 may be an effective control of metastatic EOC disease. In addition, targeting uPA or CD44 may provide additive or synergistic treatment benefits if used in combination with conventional therapeutics (chemotherapy or radiation), in particular in late-stage, metastatic, drug-resistance EOC, for which potent conventional regimens already exist.

In summary, we have shown for the first time that a coexpression of uPA and CD44 with MDR markers was found in all primary and metastatic cell lines, in most primary and matched metastatic lesions of EOC, and the overexpression of uPA, CD44 and MDR1 was significantly associated with EOC progression. The colocalisation of uPA and CD44 with MDR proteins in tumour cells and stromal cells further highlights the importance of invasive markers in the regulation of drug resistance in the progression of EOC. Our results suggest that both uPA and CD44 may be potential therapeutic targets for treating late-stage, incurable, recurrent metastatic EOC to overcome drug resistance.

## Figures and Tables

**Table 1:** The characteristics of primary EOC patients

	**EOC patients**	**Control subjects**
Number of patients	120	20
Age mean±s.d. (years)	53±18	51±14
Range	45–76	40–70
		
*Grading*		
Grade 1	18 (15%)	
Grade 2	35 (29%)	
Grade 3	67 (56%)	
		
*Clinical stage (FIGO)*	No. (%)	
I	9 (7%)	
II	25 (21%)	
III	80 (67%)	
IV	6 (5%)	
		
*Histology*		
Serous	70 (58%)	
Mucinous	23 (19%)	
Undifferentiated	11 (9%)	
Endometrioid	8 (7%)	
Clear cell	8 (7%)	
		
*Residual tumour after the first surgery*		
No	68 (57%)	
Yes	52 (43%)	
		
*Relapse*		
No	33 (28%)	
Yes	87 (72%)	
		
*Ascites*		
No	22 (18%)	
Yes	98 (82%)	

EOC=epithelial ovarian cancer; FIGO=Federation of Gynecology and Obstetrics (Creasman, 1989).

**Table 2:** Inmmunofluoresence staining intensity of uPA, CD44, MDR1 and MRP2 in EOC cell lines

	**Ovarian cancer cell line**			
	**Primary EOC cell line**		**Metastatic EOC cell line**	
**Marker**	**OVCAR-3**	**A2780**	**SKOV-3**	**OV-90**
uPA	2	2	3	2–3
CD44	2	2	3	2
MDR1	1–2	2	2	3
MRP2	2	1–2	2	2–3

*Notes:* Immunofluorescence staining scores: 0=negative; 1=weak; 2=moderate; 3=strong.

**Table 3:** uPA, CD44 and MDR1 immunoreactivity in the primary EOC and metastatic lesions

**Antigen**	**uPA (#394 MAb)**						**CD44 (MAb)**						**MDR1 (sc-1517-R Ab)**					
**Specimens**	**Immunostaining^a^ %**				**Pos^b^**	**% HEG^c^**	**Immunostaining^a^**				**%Pos^b^**	**HEG^c^**	**Immunostaining^a^**				**%Pos^b^**	**HEG^c^**
**Score**	**0**	**1+**	**2+**	**3+**			**0**	**1+**	**2+**	**3+**			**0**	**1+**	**2+**	**3+**		
PT (*n*=120)	15	16	47	42	88	74	20	26	43	31	83	62	24	20	42	34	80	64
MT (*n*=40)	4	7	16	13	90	73	6	8	14	12	85	65	7	8	14	11	83	63

a Immunostaining staining score: 0=negative; 1+=weak; 2+=moderate; 3+=strong.

b % Immunopositive tumours (score 1+ to 3+) in each subgroup;

c % of tumours ⩾score 2+ immunostaining in each subgroup (high-expression group=HEG); MT=metastatic tumours; PT=primary tumours.

**Table 4:** Clinicopathological characteristics associated with uPA, CD44 and MDR1 expression in primary EOCs

	**No. of uPA, CD44 and MDR1 intensity/total no. (%)**								
	**UPA**			**CD44**			**MDR1**		
**Variable**	**LEG**	**HEG**	*P*-value*	**LEG**	**HEG**	*P*-value*	**LEG**	**HEG**	*P*-value*
*Tumour grade*									
Low (1)	88% (16/18)	11% (2/18)	<0.0001	94% (17/18)	6% (1/18)	<0.0001	100% (18/18)	0% (0/18)	<0.0001
High (2–3)	15% (15/102)	85% (87/102)		28% (29/102)	72% (73/102)		26% (26/102)	74% (76/102)	
									
*FIGO stage*									
Low (I–II)	41% (14/34)	59% (20/34)	0.016	59% (20/34)	41% (14/34)	0.004	53% (18/34)	47% (16/34)	0.020
High (III–IV)	20% (17/86)	80% (69/86)		30% (26/86)	70% (60/86)		30% (26/86)	70% (60/86)	
									
*Histology*									
Serous	23% (16/70)	77% (54/70)	0.378	43% (30/70)	57% (40/70)	0.228	39% (27/70)	61% (43/70)	0.608
Non-serous	30% (15/50)	70% (35/50)		32% (16/50)	68% (34/50)		34% (17/50)	66% (33/50)	
									
*Residual tumour*									
No	38% (26/68)	62% (42/68)	0.004	50% (34/68)	50% (34/68)	0.003	47% (32/68)	53% (36/68)	0.007
Yes	10% (5/52)	90% (47/52)		23% (12/52)	77% (40/52)		23% (12/52)	77% (40/52)	
									
*Relapse*									
No	12% (4/33)	88% (29/33)	0.035	18% (6/33)	82% (27/33)	0.005	18% (6/33)	82% (27/33)	0.014
Yes	31% (27/87)	69% (60/87)		46% (40/87)	54% (47/87)		44% (38/87)	56% (49/87)	
									
*Ascites*									
<500 ml	9% (2/22)	91% (20/22)	0.047	18% (4/22)	82% (18/22)	0.032	18% (4/22)	82% (18/22)	0.047
⩾500 ml	30% (29/98)	70% (69/98)		43% (42/98)	57% (56/98)		41% (40/98)	59% (58/98)	

HEG=high-expression group; LEG=low-expression group.

^*^χ^2^-test, *P*<0.05 significant.
